# Prevalence of considering revision rhinoplasty in Saudi patients and its associated factors

**DOI:** 10.1186/s40902-019-0237-x

**Published:** 2019-12-10

**Authors:** Najlaa Abdulrahman Alsubeeh, Mayar Abdulsalam AlSaqr, Mohammed Alkarzae, Badi Aldosari

**Affiliations:** 1 College of Medicine, King Saud University, PO Box 2925, Riyadh, 11461 Saudi Arabia; 2 Facial Plastic Division, ENT Department, King Abdulaziz University Hospital, Riyadh, Saudi Arabia

**Keywords:** Cosmetic, Complaints, Revision rhinoplasty, Esthetic, Factors

## Abstract

**Background:**

Primary rhinoplasty outcomes may not meet individual expectations. Consequently, reoperation may be advocated to improve results. This study examines the prevalence of individuals considering revision rhinoplasty, while identifying the main cosmetic and functional complaints and factors associated.

**Methodology:**

This is a cross-sectional study conducted in Saudi Arabia using a self-reported online questionnaire distributed through social media channels. The sample included 1370 participants who were all Saudi nationals over the age of 16 who had undergone primary rhinoplasty at least 1 year prior.

**Results:**

The prevalence of individuals considering revision rhinoplasty was 44.7%. The primary reason for considering it was the desire for further esthetic improvement in an already acceptable result (50.16%). The most common cosmetic complaints subjectively reported were poorly defined nasal tip (32.35%). The most prevalent nasal function symptom was nasal obstruction (56.9%). Significant factors associated with considering revision rhinoplasty included the physician not understanding the patient’s complaints, short consultation time, low monthly income, inadequate information about the expected results, not using computer imaging to predict outcomes, lack of rapport with the surgeon, and inadequate information about the risks and complications.

**Conclusions:**

A thorough understanding of patient concerns and expectations, as well as thoughtful consideration of risk factors, may help surgeons achieve more successful outcomes and potentially reduce the incidence of revision rhinoplasties.

**Level of evidence:**

III

## Introduction

The human nose has a profound contribution to the facial esthetic [1]. It is commonly considered the most prominent feature of the face [2]. A transformative procedure in such an important area requires a great deal of attention. A rhinoplasty is a surgical procedure with a dual role: to reconstruct the shape of the nose while preserving or improving its airway function [3]. In recent years, the number of rhinoplasty procedures has increased in Saudi Arabia, currently representing 30% of all esthetic procedures in the country [4]. Patients seeking rhinoplasty have functional, esthetic, or combined defects, which may be either congenital or acquired [5]. These complaints may be regarding any of the parts of the nasal anatomy [6], which surgeons categorize into three distinct parts: cartilaginous dorsum, bony dorsum and soft tissue, or skin. Rhinoplasty is considered one of the most challenging, complex, and unpredictable cosmetic surgeries worldwide [5, 7–10]. The complexity of the procedure stems from the anatomical position of the nose, as well as distinct patient preferences. Other difficulties include the need for mastery of nasal anatomy by the surgeon, as well as simultaneously maintaining both cosmetic and functional aspects of the nose [5, 7].

The results of primary rhinoplasty may not always meet individual preoperative expectations. Consequently, reoperation may be advocated to improve surgical results [3, 8]. Research has shown that approximately 7.5–23% of subjects eventually undergo revision, or secondary rhinoplasty [6, 11–14]. Common reasons for undergoing a revision rhinoplasty include failure of the primary procedure to correct the patient’s original complaint, the desire to correct a new iatrogenic deformity after the primary rhinoplasty, and loss of personal and familial characteristics [15–17]. Secondary rhinoplasty may be technically and emotionally more challenging than primary rhinoplasty [8, 18]. Scarring from the previous procedure, loss of landmarks, and inadequate knowledge of what was previously performed pose difficulties in secondary surgeries [7, 18, 19]. Moreover, unfavorable outcomes from the previous procedure may cause emotional distress, which could affect the patient when considering a secondary rhinoplasty [1].

Concerns in primary rhinoplasty patients are often different from those in secondary rhinoplasty patients [17]. The most common complaints among patients seeking revision rhinoplasty include nasal airway obstruction, saddle nose deformity, open roof, crooked nose, dorsum asymmetry, excessive dorsal resection, residual dorsal hump, bulbous tip, tip asymmetry, drooping tip, hanging columella, alar collapse, nostril asymmetry, and wide nostrils [8, 14, 17, 20–22]. Moreover, it has been widely reported that issues with breathing functionality are common among revision patients [16, 17, 20, 23, 24]. There is controversy however, over which type of complaint is more common among patients seeking revision rhinoplasty: esthetic or functional [20, 22, 23, 25]. Some studies reported functional problems being more common [22, 23], while others demonstrated that cosmetic complaints were dominant [20, 23]. Therefore, understanding candidate concerns is of paramount importance to obtaining optimal outcomes [14].

To the best of our knowledge, there are no previous studies solely examining the prevalence of patients considering revision rhinoplasty prior to undergoing a secondary procedure, nor the causes leading such patients to considering such a procedure. This study aims to determine the prevalence of the Saudi population considering revision rhinoplasty, identifying their main cosmetic and functional complaints and possible factors associated with considering revision rhinoplasty.

## Methods

### Study design and setting

This is a cross-sectional study carried out in Saudi Arabia over a period of 5 months from January 2019 to May 2019. A self-reported online questionnaire was distributed through social media. Based on a recent bulletin published by the General Authority for Statistics in Saudi Arabia, 89.77% of the Saudi population has Internet access. Moreover, 72.54% of those use the Internet for social media [26]. This study included individuals who agreed to participate, were Saudi nationals, had undergone primary rhinoplasty at least a year prior, and were over the age of 16. Those who had undergone rhinoplasty more than once, and those who had rhinoplasty performed less than 12 months before were excluded from the study.

### Questionnaire items

The questionnaire was divided into eight parts. It began with a screening page that allowed only the respondents who met the study criteria to access the rest of the questionnaire. The next two sections inquired about sociodemographic characteristics, and whether or not the participants were considering revision rhinoplasty. The fourth section concerned the primary expressed reasons for considering revision rhinoplasty, and how serious the participants were about undergoing a revision rhinoplasty. The latter was assessed based on a 5 point Likert scale. The fifth part of the questionnaire asked participants what their esthetic concerns were for each portion of the nose. The nose was divided into the upper part, middle part, nasal tip, and other portions of the nose (columella and nostril). Images were included to clarify each part. The respondents were able to select multiple options from a defined list of choices for each part of the nose. For the upper and middle parts of the nose, the complaints were subdivided into high (hump), low, wide, narrow, crooked, and others. The complaints related to the nasal tip were subdivided into wide (bulbous), narrow (pinched), high (pointed upward), droopy (pointed downward), prominent (sticks out too far), asymmetrical, poorly defined, and others. For complaints of other portions of the nose, the following options were provided: wide nostrils, narrow nostrils, nostril asymmetry, long columella, and short columella. For each of the complaint lists, an “other” option was provided, where the participants would be able to type in their input. The sixth part of the questionnaire assessed the symptoms of nasal obstruction using the Nasal Obstruction and Septoplasty Effectiveness (NOSE) scale, which consists of 5 items [27]. The NOSE scale is a validated instrument used widely to evaluate the severity of nasal obstruction symptoms in adults. The results were recorded for each score on a scale ranging from 0 to 4. The total questionnaire score had a potential range between 0 and 20. These scores were multiplied by 5, generating a balanced scale from 0 to 100. Nasal obstruction was categorized as mild (0–25), moderate (26–50), or severe (> 50) [27]. Permission was obtained from the original author to use the NOSE scale in this study [27]. In addition, permission to use a validated Arabic version of the NOSE scale was obtained from Amer et al. [28]. The seventh and eighth parts of the questionnaire assessed possible factors in considering revision rhinoplasty. In order to ensure that the final questionnaire was understood by participants, 20 pilot participants were asked to mention any items they felt were unclear or confusing in items of language, reading ability, or comprehensibility.

### Sample size and sampling technique

The required sample size was estimated to be 386 participants. Calculation of the study sample was made based on the following formula *n* = (*Z*^2*P(1-*P*))/*d*^2; where *n* = sample size, *z* = level of confidence (2-sided 95% confidence interval), *p* = expected percentage of rhinoplasty (0.5), and *d* = precision (5%) [29, 30]. In order to reduce sampling error, the sample size of the study was increased. A simple random sample of 1370 out of 2740 (50%) participants who met the study criteria and completed the questionnaire were involved in the current study.

### Statistical analyses

Data were analyzed using Statistical Package for Social Studies (IBM Corp. Released 2013. IBM SPSS Statistics for Windows, Version 22.0. Armonk, NY: IBM Corp). Continuous variables were expressed as mean ± standard deviation and categorical variables were expressed as percentages. Chi-square test was used for categorical variables. Univariate and multivariate logistic regression were used to assess the risk factors of considering revision rhinoplasty. A *p* value was deemed statistically significant at *p* < 0 .05. Cronbach’s alpha index was applied for internal consistency reliability. Acceptable values were considered to range from 0.70 to 0.95 [31].

### Ethical approval

The present study was approved by the Institutional Review Board (IRB) of the College of Medicine, King Saud University. Informed consent was obtained from all study participants prior to commencement of the questionnaire. All information collected in the study was confidential and was not used for other purposes. Respondent anonymity was maintained as no name or identifying information were required to respond. Internet protocol addresses (IP) were immediately deleted after being used to exclude duplicated responses or multiple responses from the same individual.

## Results

Out of 4000 respondents who were approached for the study, 2740 met the inclusion criteria and enrolled, giving a response rate of 68.5%. Fifty percent (1370 out of 2740) were selected randomly for the following statistical analysis.

The majority of the study sample were female (84.38%). The mean age of the subjects was 20.9 ± 5.54 years, with a range of 17–55 years. The demographic data of the 1370 participants are summarized in Table 1.

**Table 1:** Characteristics of the participants

		*N* (1370)	%
Nationality	Saudi	1370	100.00
Age (mean, SD)		*20.9*	*5.546*
Gender	Male	214	15.62
	Female	1156	84.38
Marital status	Single	840	61.31
	Married	418	30.51
	Divorced	101	7.37
	Widow	11	0.80
Monthly income	Less than 5000	129	9.42
	Between 5000 and 10,000	306	22.34
	From 11,000 to 15,000	258	18.83
	From 16,000 to 20,000	186	13.58
	From 21,000 to 25,000	121	8.83
	26,000 to 30,000	110	8.03
	More than 30,000	260	18.98
Educational level	Illiterate	1	0.07
	Elementary	7	0.51
	Intermediate	6	0.44
	Secondary	190	13.87
	University or above	1166	85.11
Employment status	Employed	663	48.39
	Unemployed	200	14.60
	Student	393	28.69
	Retired	11	0.80
	Housewife	103	7.52
Residency	Middle region	*356*	*25.985*
	North region	*246*	*17.956*
	South region	*207*	*15.109*
	Eastern region	*287*	*20.948*
	West region	*274*	*20*

Six hundred and twelve (44.7%) of the participants were considering revision rhinoplasty. However, only 49 (8%) were seriously considering it. The most commonly expressed reasons for considering revision rhinoplasty were the desire for further esthetic improvement in an already acceptable result in 307 participants (50.16%), followed by failure to correct the original cosmetic complaints in 182 individuals (29.74%), and the development of new esthetic complaints among 152 participants (24.84%). Other primary reasons for considering revision rhinoplasty were the development of new functional complaints in 82 participants (13.40%), failure to correct the original functional complaints in 63 individuals (10.29%), the desire for further functional improvement in an acceptable result in 56 participants (9.15%), and the loss of personal and familial characteristics in 17 participants (2.78%).

The subjective cosmetic complaints for each portion of the nose among those considering revision rhinoplasty are summarized in Table 2.

**Table 2:** Esthetic concerns (nasal appearance): upper, middle, tip, other portions

	N (612)	%
Nasal appearance		
The upper portion of the nose		
Too high (hump/bump)	75	12.25
Too low (“caved-in” appearance)	65	10.62
Too wide	67	10.95
Too narrow	10	1.63
Crooked	122	19.93
No complaint in this portion of the nose	335	54.74
Other	2	0.33
Middle portion of nose		
Too high (hump/bump)	61	9.97
Too low (“caved-in” appearance)	65	10.62
Too wide	92	15.03
Too narrow	18	2.94
Crooked	180	29.41
No complaint in this portion of the nose	249	40.69
Other	1	0.16
Tip of nose		
Too wide	158	25.82
Too narrow/pinched	10	1.63
Too high (points upward)	37	6.05
Droopy (points downward)	115	18.79
Too prominent (sticks out too far)	47	7.68
Asymmetrical	191	31.21
Poorly defined	198	32.35
No complaint in this portion of the nose	88	14.38
Other	10	1.63
Other portions of the nose		
Nostrils too wide	150	24.51
Nostrils too narrow	28	4.58
Columella (soft tissue portion between nostrils) too long	121	19.77
Columella too short	28	4.58
No complaint in this portion of the nose	274	44.77
Nostril asymmetry	48	7.84
Other portions	27	4.41

The most frequently reported functional complaints were nasal obstruction (56.9%), nasal stuffiness (51%), and trouble breathing through the nose (50.2%). Other functional complaints included being unable to get enough air through the nose during exercise or exertion in 47.7%, and trouble sleeping in 37.7%.

The overall reliability score (Cronbach’s alpha) for the NOSE scale was 0.928, which demonstrates high reliability of the Arabic version of the NOSE scale. Three hundred and eighty (62.09%) had mild nasal obstruction, while 118 (19.28%) were found to have moderate nasal obstruction, and 114 participants (18.63%) had severe nasal obstruction. However, 55.4% reported intact nasal function before their primary rhinoplasty. The majority of participants seriously considering revision rhinoplasty had mild symptoms of nasal obstruction (75.61%), with a *p* value < 0.001.

Univariate analysis showed eleven significant factors that accounted for considering revision rhinoplasty. These factors were being employed, being currently or previously married, having a lower family monthly income (less than or equal to 15,000 Saudi riyals (SAR)), receiving inadequate information about nasal problems prior to the primary procedure, receiving inadequate information about expected results post-rhinoplasty, and receiving inadequate information about the risks and complications prior to the previous operation. Moreover, dissatisfaction with the rapport with the surgeon, not understanding the patient’s complaint, inadequate physician time with the patient, disuse of computer imaging in predicting rhinoplasty results, not enough clinic visits before primary rhinoplasty (i.e., one visit), and short duration of the primary consultation (less than 20 min) were significant factors related to the patient considering a revision rhinoplasty (Table 3).

**Table 3:** Univariate and multivariate logistic regression for risk factors of thinking to seek revision rhinoplasty

Risk factors		Univariate				Multivariate			
		OR	95% CI		*p* value	OR	95% CI		*p* value
			Lower	Upper			Lower	Upper	
Gender	Male	0.922	0.687	1.238	0.590				
	Female**	1.000							
Employment status	Employed	1.250	1.009	1.547	0.041*	1.020	0.786	1.324	0.879
	Unemployed**	1.000				1.000			
Family monthly income	≤ 15,000 SR	1.486	1.200	1.841	< 0.001*	1.328	1.044	1.690	0.021*
	> 15,000 SR**	1.000				1.000			
Marital status	Not single	1.421	1.142	1.768	0.002*	1.164	.861	1.573	0.325
	Single**	1.000				1.000			
Educational level	Secondary or less	0.846	0.626	1.144	0.277				
	University and above**	1.000							
Receiving adequate information about your nasal problem before undergoing primary rhinoplasty?	Yes**	1.000			< 0.001*	1.000			0.546
	No	2.855	2.218	3.675		1.107	0.795	1.543	
Receiving adequate information about expected results after the operation?	Yes**	1.000			< 0.001*	1.000			< 0.001*
	No	3.605	2.872	4.525		2.094	1.522	2.881	
Receiving adequate information about risks and complications of the previous operation?	Yes**	1.000			< 0.001*	1.000			0.004*
	No	1.883	1.513	2.344		0.649	0.480	0.877	
Satisfaction with the rapport between patient and surgeon	Yes**	1.000			< 0.001*	1.000			0.021*
	No	3.579	2.818	4.546		1.472	1.059	2.046	
Feeling that surgeon is comprehends patient problems	Yes**	1.000			< 0.001*	1.000			0.001*
	No	3.816	2.987	4.875		1.739	1.268	2.386	
Feeling that surgeon spent adequate time with patient	Yes**	1.000			< 0.001*	1.000			0.001*
	No	3.280	2.626	4.098		1.675	1.241	2.260	
Surgeon has shown patient a computer imaging of predicted result before primary rhinoplasty	Yes**	1.000			< 0.001*				< 0.001*
	No	2.219	1.718	2.867		1.763	1.316	2.363	
Frequency of visiting surgeon before undergoing primary rhinoplasty	One visit	1.930	1.279	2.913	0.001*	1.241	0.777	1.983	0.367
	Two visits	1.261	0.833	1.909	0.274	1.107	0.696	1.761	0.667
	Three visits	1.114	0.706	1.757	0.643	1.187	0.714	1.974	0.509
	4 visit or more**	1.000							
Experienced complications following primary surgery	Yes	1.183	0.902	1.552	0.224				
	No**	1.000							
Presence of psychiatric disease	Yes	0.933	0.717	1.214	0.604				
	No**	1.000							
Duration of consultations	≤ 20 min	2.240	1.711	2.931	< 0.001*	1.267	0.920	1.744	0.147
	> 20 min*	1.000				1.000			

*Significant *p* value

**Used as a reference

A multivariate logistic regression incorporated all significant results from the univariate logistic regression to determine independently significant factors associated with considering revision rhinoplasty (Table 3). The most significant factors associated with considering revision rhinoplasty were receiving inadequate information about the expected results post-rhinoplasty (OR = 2.094; 95% CI 1.522–2.881, *p* value < 0.001), followed by disuse of computer imaging in predicting post-operative results before the previous rhinoplasty (OR = 1.763; 95% CI 1.316–2.363, *p* value = < 0.001), not understanding the patient’s complaints (OR = 1.739; 95% CI 1.268–2.386, *p* value = 0.001), inadequate physician time with the patient (OR = 1.675; 95% CI 1.241–2.260, *p* value = 0.001), dissatisfaction with the rapport with the surgeon (OR = 1.472; 95% CI 1.059.–2.046, *p* value = 0.021), a family monthly income less than or equal to 15,000 SAR (OR = 1.328; 95% CI 1.044–1.690, *p* value = 0.021), and receiving inadequate information about the risks and complications of the operation (OR = 1.000; 95% CI 0.480–0.877, *p* value = 0.004) (Table 3).

Table 4 shows the comparison outcomes between those who are considering revision rhinoplasty and those who are not in respect to its possible affecting factors. Out of all sociodemographic factors, social status, monthly income, and employment status showed a statistically significant difference between the two groups. The chi-squared test showed a statistically significant association between being single and not considering revision rhinoplasty (*p* = 0.009). However, having a low monthly income was significantly associated with considering a revision rhinoplasty (*p* < 0.001). Also, being employed was associated with considering re-operation (*p* = 0.0004). Other factors that were associated significantly with not considering revision rhinoplasty included receiving adequate information (regarding nasal problems, expected results, risks, and complications) (*p* < 0.0001), satisfaction with the rapport with the surgeon (*p* < 0.0001), and feeling that surgeon has comprehended patient’s nasal problems (*p* < 0.001). On the other hand, factors that were associated with considering revision rhinoplasty included a lower number of clinic visits before primary operation (*p* < 0.001), spending inadequate time during the consultation (*p* < 0.001) and not using computer imaging of the predicted result before undergoing primary rhinoplasty (*p* < 0.001).

**Table 4:** Possible factors affecting patient to consider (think) to seek revision rhinoplasty

		Thinking (considering) to seek revision		Not thinking (Not considering) to seek revision		*χ*^2^	*p* value
		*N*	%	*N*	%		
Overall		612	44.67	758	55.33		
Gender	Male	92	15.03	122	16.09	0.29	0.590
	Female	520	84.97	636	83.91		
Social status	Single	347	56.70	493	65.04	11.58	0.009*
	Married	203	33.17	215	28.36		
	Divorced	56	9.15	45	5.94		
	Widow	6	0.98	5	0.66		
Monthly income	Less than 5000	83	13.56	46	6.07	33.63	0.001*
	Between 5000 and 10,000	146	23.86	160	21.11		
	From 11,000 to 15,000	114	18.63	144	19.00		
	From 16,000 to 20,000	86	14.05	100	13.19		
	From 21,000 to 25,000	49	8.01	72	9.50		
	26,000 to 30,000	43	7.03	67	8.84		
	More than 30,000	91	14.87	169	22.30		
Educational level	Illiterate	0	0.00	1	0.13	2.52	0.641
	Elementary	2	0.33	5	0.66		
	Intermediate	3	0.49	3	0.40		
	Secondary	79	12.91	111	14.64		
	University or above	528	86.27	638	84.17		
Employment status	Employed	315	51.47	348	45.91	15.19	0.004*
	Unemployed	90	14.71	110	14.51		
	Student	150	24.51	243	32.06		
	Retired	2	0.33	9	1.19		
	Housewife	55	8.99	48	6.33		
Receiving adequate information about nasal problem before undergoing primary rhinoplasty	Yes	390	63.7	632	83.4	69.01	< 0.001*
	No	222	36.3	126	16.6		
Receiving adequate information about expected results after the operation	Yes	267	43.6	558	73.6	127.11	< 0.001*
	No	345	56.4	200	26.4		
Receiving adequate information about risks and complications of the previous operation	Yes	314	51.3	504	66.5	32.45	< 0.001*
	No	298	48.7	254	33.5		
Satisfaction with the rapport between patient and surgeon	Yes	325	53.1	608	80.2	114.53	< 0.001*
	No	287	46.9	150	19.8		
Feeling that surgeon is comprehends patient problems	Yes	335	54.7	623	82.2	121.34	< 0.001*
	No	277	45.3	135	17.8		
Surgeon spent adequate time with patient	Yes	215	35.1	485	64.0	112.81	< 0.001*
	No	397	64.9	273	36.0		
Duration of the consultation before undergoing primary rhinoplasty	Less than 10 min	207	33.8	160	21.1	53	< 0.001*
	10–15 min	214	35.0	229	30.2		
	16–20 min	96	15.7	148	19.5		
	21–25 min	30	4.9	73	9.6		
	25–30 min	33	5.4	78	10.3		
	More than 30 min	32	5.2	70	9.2		
Surgeon has shown patient a computer imaging of predicted result before primary rhinoplasty	Yes	110	18.0	248	32.7	38.13	< 0.001*
	No	502	82.0	510	67.3		
Experiencing complications following previous surgery	Yes	124	20.3	134	17.7	1.48	0.224
	No	488	79.7	624	82.3		
Frequency of visiting surgeon before undergoing your primary rhinoplasty	One visit	273	44.6	250	33.0	20.82	< 0.001*
	Two visits	204	33.3	286	37.7		
	Three visits	92	15.0	146	19.3		
	4 visit or more	43	7.0	76	10.0		
Presence of psychiatric disease	Other	0	0.00	4	0.53	5.711	0.574
	Depression	55	8.99	72	9.50		
	Anxiety	23	3.76	34	4.49		
	Body dysmorphic disorder	6	0.98	6	0.79		
	Personality trait abnormality	9	1.47	10	1.32		
	Obsessive compulsive disorder	5	0.82	8	1.06		
	Anorexia nervosa	3	0.49	1	0.13		
	I am not diagnosed with any psychiatric disease	511	83.50	623	82.19		

*Significant *p* value

## Discussion

This study measured the prevalence of patients considering undergoing revision rhinoplasty, as well as identified their main cosmetic and functional complaints, their primary reasons for considering the surgery, and possible factors associated with considering revision rhinoplasty. In our study, 44.7% of participants who had undergone a primary rhinoplasty were considering revision rhinoplasty. This rate is much higher than the rates of patients who had actually undergone revision rhinoplasty, which has been reported to be between 7.5 and 23% [8, 11–14, 32]. However, only 8% of our study population were seriously considering revision rhinoplasty, which is relatively closer to revision rhinoplasty rates that have been previously reported [12, 3].

In the current study, the most frequently presented motivation for considering revision rhinoplasty was the desire for further esthetic improvement in an already acceptable result (50.16%), followed by failure to correct the original cosmetic complaints (29.74%), and the development of new esthetic complaints (24.84%). In contrast, Constantian MB et al. revealed that the most common reasons for undergoing revision rhinoplasty were the development of a nonexistent deformity (41%), failure to correct the primary cosmetic deformity (33%), and the loss of ethnic or personal characteristics (15%), with only 10% wanting further improvement in an already acceptable result [16]. This contrast could be due to the differences between the two study populations. The population in Constantian MB et al’s study was responding retrospectively after they had performed revision rhinoplasty, whereas our participants were still considering the procedure. It is important to note that not all of those considering revision are necessarily going to seek re-operation. In addition, the ability of our participants to select more than one reason for considering revision rhinoplasty in the questionnaire might play a role in this variation. However, the aforementioned findings indicate that the vast majority of our participants were considering revision rhinoplasty due to esthetic concerns. Prior studies demonstrate similar results, where cosmetic complaints were more common than functional complaints among revision rhinoplasty patients [16, 20, 23, 25].

Surgeons should understand the cosmetic and functional concerns that their patients consider most troublesome. The results of the present study show that the most prevalent esthetic complaint concerning the upper and middle portion of the nose is crookedness. Other studies have also found that 36–38% of patients admitted for re-operation are more likely to have crookedness as their primary esthetic complaint [14, 25].

Concerning the tip of the nose, the most common complaints reported subjectively by participants were poorly defined nasal tip (32.35%), followed by asymmetrical nasal tip (31.21%), and wide (bulbous) nasal tip (25.82%). Such complaints have also been reported by secondary rhinoplasty patients in multiple studies [8, 14, 20, 24]. This is perhaps attributable to the technical challenges of tip refinement maneuvers and the failure to address the nasal tip at the time of operation.

The most frequently stated cosmetic concerns of the columella and nostrils were having a long columella (19.77%) and wide nostrils (24.51%). A study conducted by Kathy Yu et al., showed similar findings [24]. These cosmetic concerns shed light on the importance of preoperative deformity analysis of all aspects of the nose.

We found that 37.91% of those considering revision rhinoplasty complained of either moderate or severe nasal obstruction symptoms as assessed subjectively using the NOSE scale. Vian HN et al. also found that 37.2% of patients submitted for revision rhinoplasty have obstructive respiratory concerns [20]. However, Kathy Yu et al. showed an even higher percentage of patients experiencing nasal obstruction complaints (62%) [24]. Differences in the prevalence of obstructive symptoms may be attributed to contrasts in surgeon vigilance, and ultimately paying attention to functional concerns prior to operating on a patient. In our study, the top three nasal function symptoms reported by those considering revision rhinoplasty were nasal obstruction (56.9%), followed by nasal stuffiness (51%), then trouble breathing through the nose (50.2%). Similarly, Kathy Yu et al. demonstrated that the sensation of nasal blockage ranked to be the main functional symptoms among revision rhinoplasty patients [24].

Certain factors can negatively affect the outcomes of primary rhinoplasty leading a patient to consider a revision rhinoplasty. We were able to identify seven independent factors associated with considering this procedure. Receiving inadequate information about the expected results was the most significant predictor of considering revision rhinoplasty among our sample. Unrealistic expectations have been linked to negative outcomes of rhinoplasty [33]. Thus, it is important to acknowledge patients’ expectations carefully and clarify all expected outcomes to ensure delivering a realistic expectation.

The second associated factor for considering revision rhinoplasty was not using computer imaging, or morphing, in predicting the postoperative result before the primary rhinoplasty. Despite the role of computer imaging as a tool to enhance communication between the patient and surgeon, the accuracy of the image is directly related to the imaging skills of the surgeon, limiting its reliability [34–36]. Therefore, it is vital to use imaging with caution and educate patients accordingly [34].

The third, fourth, and fifth predictive factors were the surgeon’s lack of understanding the patient’s problems, spending inadequate time with the patient, and patient dissatisfaction with the relationship with the surgeon. This emphasizes the impact of the surgeon’s encounter with the patient, and how that may influence consideration of revision rhinoplasty. Moreover, this highlights the value of establishing a good surgeon-patient relationship, which cannot be fulfilled without good communication skills. Proper communication consequently enhances the ability to address patients’ concerns, as well as increase the quality of the clinical consultation.

Our study did not reveal a significant correlation between almost all sociodemographic factors (gender, marital status, educational level, employment status) and considering a revision rhinoplasty, except for family income. Lower family monthly income (i.e., those with a monthly income of ≤ 15,000 Saudi riyals,) was the sixth significant predictor found to be associated with participants’ consideration of revision rhinoplasty. We hypothesize that a lower budget available for performing a rhinoplasty may encourage patients to choose their surgeons according to the overall cost of the procedure, without taking into account the surgeon’s level of qualification. As a result, patients may not exactly obtain their desired outcome, and may wish for further improvement.

Surprisingly, receiving adequate information about the risks and complications of the procedure was ranked as the seventh predictor associated with considering revision rhinoplasty. This might be attributed to the fact that clear explanation of all potential surgical benefits, risks and complications involved would help in dispelling misconceptions and provide thorough knowledge of the nature of a rhinoplasty. Consequently, the patient may feel under less pressure to proceed with that operation again, especially due to prior self-experience of rhinoplasty and their familiarity with it.

Limitations of this study include that the identified esthetic and functional nasal concerns were subjectively reported by the participants, and were not objectively confirmed by surgeons. However, images were included in the questionnaire in an attempt to clarify nasal anatomy for better reporting of esthetic problems by the participants. Strengths of this study include the sample size, which is larger than in other studies in the literature. Second, our sample was discrete, as we targeted those who were only considering revision rhinoplasty, and not those who had actually undergone revision rhinoplasty. We were able to identify their main cosmetic and functional complaints, their primary reason to consider revision surgery, and the factors associated with considering revision rhinoplasty. Third, multiple surgeon and patient-related factors were assessed to predict their possible association with considering revision rhinoplasty.

## Conclusion

Rhinoplasty remains one of the most complex and challenging operations in plastic surgery. Our study showed a high prevalence of patients considering revision rhinoplasty. Our findings show that cosmetic complaints were more common than functional complaints, and that the primary reason for seeking revision rhinoplasty was the desire for further esthetic improvement in an already acceptable result. This emphasizes the importance of detailed assessment and clarification concerning the patient’s expectations and actual surgical possibilities. Proper communication, listening to patients, and thoughtful consideration of risk factors associated with considering revision rhinoplasty should lead to more successful outcomes.

## Data Availability

The data supporting our finding can be provided when needed.

## Abbreviations

NOSE: Nasal Obstruction and Septoplasty Effectiveness scale
SAR: Saudi riyals
